# Efficacy and safety of edoxaban versus enoxaparin for the prevention of venous thromboembolism following total hip arthroplasty: STARS J-V

**DOI:** 10.1186/s12959-015-0057-x

**Published:** 2015-08-12

**Authors:** Takeshi Fuji, Satoru Fujita, Yohko Kawai, Mashio Nakamura, Tetsuya Kimura, Masayuki Fukuzawa, Kenji Abe, Shintaro Tachibana

**Affiliations:** Department of Orthopaedic Surgery, Japan Community Healthcare Organization Osaka Hospital, 4-2-78, Fukushima, Fukushima-ku, Osaka 553-0003 Japan; Department of Orthopaedic Surgery, Takarazuka Daiichi Hospital, 19-5 Kogetsu-cho, Takarazuka, 665-0832 Japan; International University of Health and Welfare, 8-10-16 Akasaka Minato-ku, Tokyo, 107-0002 Japan; Department of Clinical Cardiovascular Research, Mie University Graduate School of Medicine, 2-174 Edobashi, Tsu, 514-8507 Japan; Clinical Planning Department, Daiichi Sankyo Co. Ltd, 1-2-58, Hiromachi, Shinagawa-ku, Tokyo, 140-8710 Japan; Clinical Execution Department, Daiichi Sankyo Co. Ltd, 1-2-58, Hiromachi, Shinagawa-ku, Tokyo, 140-8710 Japan; Clinical Data & Biostatistics Department, Daiichi Sankyo Co. Ltd, 1-2-58, Hiromachi, Shinagawa-ku, Tokyo, 140-8710 Japan; Department of Orthopaedic Surgery, Mishuku Hospital, 5-33-12 Shimomeguro, Meguro-ku, Tokyo, 153-0051 Japan

**Keywords:** Edoxaban, Enoxaparin, Total hip arthroplasty, Factor Xa, Thromboprophylaxis

## Abstract

**Background:**

In the absence of thromboprophylaxis, patients undergoing total hip arthroplasty (THA) are at increased risk for venous thromboembolism (VTE). The objective of this study was to compare the efficacy and safety of edoxaban with enoxaparin for the prevention of VTE after THA in Japan.

**Methods:**

This was a phase 3, double-blind, double-dummy, noninferiority study. Patients undergoing elective, unilateral primary THA were randomized to receive edoxaban 30 mg once daily (*n* = 307) or enoxaparin 2000 IU (equivalent to 20 mg) twice daily (*n* = 303) for 11 to 14 days. The primary efficacy endpoint was the incidence of VTE. Safety endpoints included the incidence of major or clinically relevant nonmajor (CRNM) bleeding.

**Results:**

The incidence of VTE, based on venography and clinical surveillance, was 2.4 % in the edoxaban group and 6.9 % in the enoxaparin group (*P* <0.001). The absolute difference in the incidence of VTE was −4.5 % (95 % confidence interval [CI]: −8.6, −0.9), which was within the noninferiority margin set at 8 % for the difference and established the noninferiority of edoxaban to enoxaparin. Since the upper limit of the 95 % CI of the absolute difference was less than 0 %, the superiority of edoxaban over enoxaparin was demonstrated. The incidence of major or CRNM bleeding was 2.6 % in the edoxaban group and 3.7 % in the enoxaparin group (*P* = 0.475).

**Conclusions:**

Oral edoxaban 30 mg once daily was superior to subcutaneous enoxaparin 2000 IU twice daily in the prevention of VTE following THA without increasing the risk for major or CRNM bleeding.

## Background

Venous thromboembolism (VTE), comprising deep vein thrombosis (DVT) and pulmonary embolism (PE), is a leading cause of morbidity and mortality [1]. In the absence of thromboprophylaxis, 40 % to 60 % of patients undergoing total hip arthroplasty (THA) or total knee arthroplasty (TKA) will develop venographic evidence of DVT, and approximately 1 in 300 THA patients will experience a fatal PE [2]. The morbidity and mortality associated with VTE strongly support primary thromboprophylaxis in THA patients [3, 4].

In Japan, the standard of care for thromboprophylaxis following THA is the low-molecular weight heparin (LMWH) enoxaparin 2000 IU twice daily, initiated 24 to 36 h after surgery and continued for 14 days [4–6]. Fondaparinux, a selective factor Xa inhibitor administered by subcutaneous injection, is another potent anticoagulant approved for the prevention of VTE in Japanese patients undergoing THA or TKA [7].

Edoxaban, a direct, once-daily factor Xa inhibitor [8], is a new oral anticoagulant that has been studied for the prevention of VTE [9, 10]. In phase 2 studies of patients undergoing elective THA [10] or TKA [9], edoxaban demonstrated significant dose-related reductions in VTE compared with dalteparin and placebo, respectively. Another phase 2 study in patients following THA demonstrated no significant difference in the incidence of VTE for patients who received edoxaban compared with enoxaparin [11]. There was a low incidence of major or clinically relevant nonmajor (CRNM) bleeding observed with edoxaban in these studies. Results from the phase 2 studies in patients following THA led to the initiation of the phase 3 Studying Thrombosis After Replacement Surgery J-V (STARS J-V) trial. The objective of the STARS J-V trial was to compare the efficacy and safety of edoxaban 30 mg once daily with enoxaparin 2000 IU (equivalent to 20 mg) twice daily in Japanese patients for the prevention of VTE following elective, unilateral, primary THA.

## Materials and methods

### Study design

This was a randomized, double-blind, double-dummy, parallel-group, enoxaparin-controlled, multicenter phase 3 trial (ClinicalTrials.gov Identifier: NCT01181167) of edoxaban in patients undergoing unilateral THA (excluding revision THA). The study protocol, information for patients, and informed consent forms were reviewed and approved by an institutional review board, and the study was conducted in accordance with the Declaration of Helsinki and Good Clinical Practice guidelines.

**Fig. 1:**
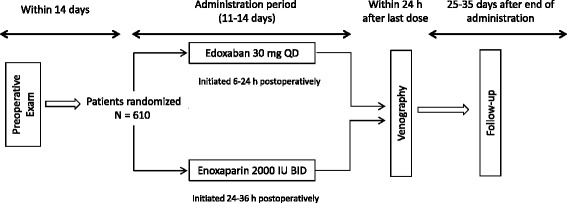
Study design. BID, twice a day; IU, international unit; QD, once daily

Patients were randomized 1:1 to receive oral edoxaban 30 mg once daily or subcutaneous enoxaparin 2000 IU twice daily for 11 to 14 days. Treatment with edoxaban (or edoxaban placebo) was administered within 6 to 24 h after surgery and once daily each morning thereafter; enoxaparin injection (or enoxaparin placebo injection) was administered 24 to 36 h after surgery and twice daily from the following day onward (Fig. 1). The enoxaparin dosing regimen was set according to the approved regimen for enoxaparin sodium in Japan. Patients were permitted to use mechanical physiotherapy, which included elastic stockings or intermittent pneumatic compression of the sole of the foot or of the lower legs and thigh. Venography of the lower limbs was performed within 24 h after either the end of treatment or after the last dose of study drug when treatment was discontinued, or within 96 h for reasons such as difficulty in establishing an intravenous line. Investigations, observations, examinations, and urine and blood sample collection (for urinalysis, edoxaban plasma concentration measurements, assessment of pharmacodynamic [PD] indices, and hematology tests) were performed at presurgical evaluation, pretreatment (postsurgery), on day seven, and on the completion day of treatment. Follow-up examination was performed at 25 to 35 days after the last dose of study drug. The occurrence of thromboembolic events, bleeding, and all other adverse events (AEs) were recorded throughout the study. If applicable, site, duration, and total time of physiotherapy use were recorded.

### Selection of study population

Patients 20 to 85 years of age undergoing unilateral THA were included. Presurgical exclusion criteria included risk for bleeding (eg, history of intracranial bleeding, comorbid gastrointestinal bleeding or peptic ulcer within the previous 3 months, or prothrombin time [PT] prolongation above the upper limit of the normal [ULN]); risk for thromboembolism (eg, history of symptomatic DVT or PE, coagulation disease at high risk for thromboembolism, or history of lower limb fracture within the previous 6 months); previous TKA; expected joint replacement of the other lower limb at the same time of anesthesia; weight <40 kg; severe renal impairment (creatinine clearance [CL_CR_] of <30 mL/min) [12]; evidence of hepatic dysfunction (serum aspartate aminotransferase [AST] or serum alanine aminotransferase [ALT] levels ≥2 times the ULN or total bilirubin ≥1.5 times the ULN); previous treatment with edoxaban; and current antithrombotic therapy for another complication. Postsurgical exclusion criteria included abnormal bleeding from the puncture site during spinal anesthesia, need for repeat surgery before the start of study treatment, abnormal or excessive bleeding experienced during surgery, and inability to take oral medication. All patients provided written informed consent prior to initiation of any study-related procedures.

Patients discontinued treatment if they developed symptomatic DVT, PE, or major bleeding; had evidence of hepatic dysfunction; had persistent systolic blood pressure of >180 mm Hg or diastolic blood pressure of >110 mm Hg; required anticoagulant drugs, antiplatelet drugs, thrombolytic drugs, or drugs that might affect thrombus formation; needed postsurgical epidural anesthesia; experienced an AE that the investigator or subinvestigator judged was sufficient for study discontinuation; voluntarily requested to withdraw; were found to be ineligible for continued participation in the study for any reason; or if the investigator judged that the study should be discontinued for the patient.

### Study endpoints

The primary efficacy endpoint was the incidence of VTE from the start of treatment to venography at the end of treatment, including asymptomatic DVT and symptomatic PE or DVT. Secondary efficacy endpoints were the incidence of symptomatic DVT, proximal DVT, symptomatic PE, or VTE-related death; the incidence of asymptomatic or symptomatic DVT; the incidence of symptomatic or proximal DVT; the incidence of symptomatic PE; the incidence of VTE-related deaths; and the incidence of all-cause deaths.

Safety endpoints included the incidences of major bleeding; CRNM bleeding; major or CRNM bleeding; any bleeding (major, CRNM, and minor); and minor bleeding events from the start of treatment to the day after the end of treatment. Major bleeding was defined as fatal bleeding; clinically overt bleeding accompanied by a decrease in hemoglobin of >2 g/dL; clinically overt bleeding requiring hemotransfusion with more than four units of blood (1 unit = approximately 200 mL); retroperitoneal, intracranial, intraocular, or intrathecal bleeding; or bleeding requiring repeat surgery. CRNM bleeding was bleeding that was not characterized as major, but corresponded to any of the following: hematoma ≥5 cm in long diameter, epistaxis or gingival bleeding in the absence of external factors and lasting ≥5 min, gastrointestinal bleeding, gross hematuria persistent after 24 h of onset, or any other bleeding deemed clinically significant by the investigator. Minor bleeding was any bleeding event (including a positive occult blood reaction) that was not considered a major or CRNM event. Additional safety endpoints were the incidences of AEs and adverse drug reactions (ADRs).

Thromboembolic events were assessed by the Thromboembolic Event Assessment Committee, which made the final assessment of thromboembolic events based on blinded copies of the test results (radiographic films, *etc.*) collected from each study site. Bleeding events were assessed by the Bleeding Event Assessment Committee, which was responsible for the final assessment of bleeding events. Results of the assessments by these committees were defined as the final evaluation.

Edoxaban plasma concentrations were measured by liquid chromatography-tandem mass spectrometry; the lower limit of quantification was 0.764 ng/mL.

### Statistical analysis

The primary analyses for the efficacy endpoints were performed for the full analysis set (FAS) and per-protocol set (PPS). The FAS was defined as all enrolled patients who received ≥1 dose of study drug. Patients who did not develop symptomatic DVT or PE, and in whom venography was not appropriately performed, were excluded from the FAS. The PPS included patients in the FAS but excluded those with violations to the inclusion/exclusion criteria, those who received prohibited concomitant medications or therapies, or whose study treatment compliance rate was <80 %. Safety analyses were performed for the safety analysis set (SAS), defined as all patients who received ≥1 dose of study drug. The pharmacokinetic (PK) analysis set included patients in the PPS with valid plasma drug concentration measured at ≥1 time points.

For all comparisons of the primary, secondary, and safety endpoints, a paired comparison was performed between treatment groups using the *χ*^2^ test, with a two-sided significance level of 0.05 and a two-sided confidence coefficient of 0.95. In the primary efficacy analysis, the incidence of the primary endpoint and 95 % confidence intervals (CIs) were calculated for each treatment group. The difference in the incidence of the primary endpoint between edoxaban and enoxaparin and its corresponding 95 % CI were then calculated using the Farrington-Manning method to test for noninferiority [13]. The noninferiority margin was set at 8 % for the difference in the primary endpoint between the two groups, with a 1-sided significance level of 0.025. This noninferiority margin was chosen as it was less than half of the reduction in VTE incidence, estimated to be at least approximately 18 % based on the incidence of VTE in Japanese patients undergoing THA (27.3 %; 95 % CI 22.2, 32.9) given in the “Japanese Guideline for the Prevention of Venous Thromboembolism” [14] and the incidence of VTE with twice-daily enoxaparin 20 mg used in the phase 2b study of patients undergoing THA (4.1 %) [15]. If noninferiority was established, edoxaban would be considered superior to enoxaparin if the upper limit of the 95 % CI calculated for the difference in the primary endpoint between the two groups was below 0 %. Similar analyses were performed for the secondary efficacy endpoints.

The incidence of major bleeding events and corresponding 95 % CIs during the treatment period were calculated for each treatment group. The difference in the incidences of major bleeding between the edoxaban and enoxaparin groups and its 95 % CI were also calculated. Similar analyses were performed for CRNM bleeding, major or CRNM bleeding, minor bleeding, and any bleeding events.

The overall incidences of AEs and ADRs and their 95 % CIs were calculated by treatment group. Hepatic dysfunction was classified by the extent of the increase of liver function parameters above the ULN, and the frequency of abnormal changes was summarized.

The number of patients necessary to verify noninferiority was calculated to be 235 patients per treatment group, providing ≥90 % power assuming a VTE incidence of 6 %. Assuming that the proportion of patients excluded from analyses would be approximately 20 %, the planned number of patients was set at 600 (300/group).

## Results

### Patient disposition

**Fig. 2:**
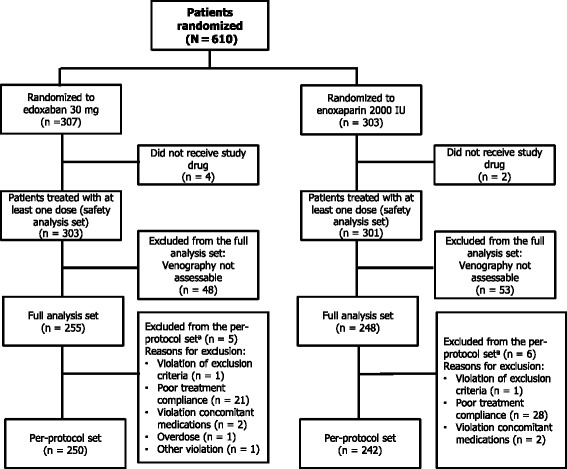
Patient disposition. IU, international unit

Between May 2009 and January 2010, 610 patients were randomized: 307 patients to edoxaban and 303 to enoxaparin (Fig. 2). Of the randomized patients, 604 received ≥1 dose of medication and were included in the SAS. Venography was unassessable in 101 patients (48 and 53 in the edoxaban and enoxaparin groups, respectively), resulting in 503 patients included in the FAS. During the study period, 23 patients in the edoxaban group and 32 patients in the enoxaparin group discontinued treatment. In both groups, the most common reasons for discontinuation were AEs and voluntary patient withdrawal. In the edoxaban group, nine patients withdrew due to AEs, six patients voluntarily withdrew, three patients were found to be ineligible, and the investigator judged withdrawal was appropriate for two patients. In the enoxaparin group, ten patients withdrew due to AEs, eight patients voluntarily withdrew, and the investigator judged withdrawal was appropriate for three patients. There were no clinically relevant differences in baseline characteristics between the treatment groups (Table 1). Mean age was 62.8 years and mean body weight was 57.4 kg. Overall, most patients were female (86 %), <75 years of age (87 %), weighed <60 kg (63 %), and had a CL_CR_ of ≥80 mL/min (59 %). Mean treatment duration for both groups was 12.5 days. Physiotherapy was used in at least 80 % of patients and was balanced between groups.

**Table 1:** Baseline characteristics

**Characteristic**	Edoxaban 30 mg QD (*n* = 255)	Enoxaparin 2000 IU BID (*n* = 248)
**Sex**		
Female	220 (86.3)	212 (85.5)
**Age (y)**		
Mean ± SD	62.8 ± 9.61	62.8 ± 9.72
<75	220 (86.3)	217 (87.5)
≥75	35 (13.7)	31 (12.5)
**Weight (kg)**		
Mean ± SD	57.7 ± 9.72	57.0 ± 9.60
<60	160 (62.7)	157 (63.3)
≥60	95 (37.3)	91 (36.7)
**BMI**, mean ± SD	24.5 ± 3.52	24.2 ± 3.60
**CL**_**CR**_ (mL/min)		
Mean ± SD	89.6 ± 29.58	88.9 ± 26.49
≥80	148 (58.0)	150 (6)
≥50 to <80	93 (36.5)	91 (36.7)
<50	14 (5.5)	7 (2.8)
**History of artificial joint replacement of the lower limbs**	39 (15.3)	35 (14.1)
**Duration from the end of surgery to the start of study treatment (h)**		
≥12 to <18	16 (6.3)	-
≥18 to ≤24	239 (93.7)	-
≥24 to <30	-	170 (68.5)
≥30 to <36	-	78 (31.5)
Hour:minute, Mean ± SD	21:40 (1:54)	28:37 (3:00)
**Physiotherapy** ^a^		
Intermittent pneumatic compression therapy (foot sole)	137 (53.7)	135 (54.4)
Intermittent pneumatic compression therapy (lower legs and thigh)	120 (47.1)	114 (46.0)
Elastic stockings	209 (82.0)	201 (81.0)
**Concomitant NSAIDs**	250 (98.0)	243 (98.0)
**Treatment duration (days)**		
Mean ± SD	12.5 (1.4)	12.5 (1.5)

Data presented as n (%) unless otherwise stated. BID, twice daily; BMI, body mass index; CL_CR_, creatinine clearance; IU, international unit; NSAIDs, non-steroidal anti-inflammatory drugs; QD, once daily; SD, standard deviation

^a^Patients could be using more than one mode of physiotherapy

### Outcome measures

The incidence of VTE is shown in Table 2. All thromboembolic events were asymptomatic DVT, as neither symptomatic DVT nor PE developed in either treatment group. In the FAS, the primary efficacy outcome occurred in 2.4 % (6/255) of patients receiving edoxaban and in 6.9 % (17/248) receiving enoxaparin, resulting in a relative risk reduction of 65.7 % (*P* <0.001). The absolute difference for the primary efficacy endpoint was −4.5 % (95 % CI: −8.6, −0.9) and was within the noninferiority margin, thereby establishing that edoxaban was noninferior to enoxaparin for the prevention of VTE. As the upper limit of the 95 % CI of the absolute difference was less than 0 %, the superiority of edoxaban over enoxaparin was demonstrated.

**Table 2:** Incidence of thromboembolic events

Outcome	Edoxaban 30 mg QD (*n* = 255)		Enoxaparin 2000 IU BID (*n* = 248)		Absolute difference % (95 % CI)
	n (%)	95 % CI	n (%)	95 % CI	
Primary efficacy (any VTE)	6 (2.4)	1.1, 5.0	17 (6.9)	4.3, 10.7	−4.5 (−8.6, −0.9)^a,b^
Asymptomatic DVT					
Total^c^	6 (2.4)	1.1, 5.0	17 (6.9)	4.3, 10.7	−4.5 (−8.6, −0.9)
Proximal	1 (0.4)	0.1, 2.2	2 (0.8)	0.2, 2.9	−0.4 (−2.5, 1.5)
Distal	6 (2.4)	1.1, 5.0	16 (6.5)	4.0, 10.2	−4.1 (−8.1, −0.6)
Symptomatic DVT	0 (0.0)	0.0, 1.5	0 (0.0)	0.0, 1.5	0.0
Symptomatic PE	0 (0.0)	0.0, 1.5	0 (0.0)	0.0, 1.5	0.0
VTE-related death	0 (0.0)	0.0, 1.5	0 (0.0)	0.0, 1.5	0.0
Symptomatic DVT, proximal DVT, symptomatic PE, or VTE-related death	1 (0.4)	0.1, 2.2	2 (0.8)	0.2, 2.9	−0.4 (−2.5, 1.5)

BID, twice daily; CI, confidence interval; DVT, deep vein thrombosis; IU, international unit; PE, pulmonary embolism; QD, once daily; VTE, venous thromboembolism

^a^ *p* <0.001 as calculated by the noninferiority Farrington-Manning test; ^b^ *p* = 0.0157 as calculated by the superiority Farrington-Manning test; ^c^one patient had both a proximal and a distal DVT

Among patients who utilized physiotherapy, there was a significant reduction in the incidence of VTE with use of elastic stockings in the edoxaban group compared with the enoxaparin group (1.9 % [4/209] vs 7.5 % [15/201], respectively; *P* = 0.004). The incidences of VTE as assessed by venography were similar between the edoxaban and enoxaparin groups with intermittent pneumatic compression therapy of the sole of the foot (2.9 % [4/137] vs 4.4 % [6/135], respectively) and of the lower legs and thigh (4.2 % [5/120] vs 5.3 % [6/114], respectively).

The incidence of symptomatic DVT, proximal DVT, symptomatic PE, or VTE-related death was 0.4 % (1/255) in the edoxaban group and 0.8 % (2/248) in the enoxaparin group (Table 2). All events were asymptomatic, proximal DVT. The absolute difference in the incidence was −0.4 % (95 % CI: −2.5, 1.5), with no clear differences between the two groups (Table 2). No VTE-related deaths occurred during the study. Primary and secondary efficacy outcomes for the PPS population paralleled those of the FAS.

### Safety

The incidence of major bleeding was 0.7 % and 2.0 % in the edoxaban and enoxaparin groups, respectively, for an absolute difference of −1.3 % (*P* = 0.176) (Table 3). Clinically relevant nonmajor bleeding was reported by 2.0 % and 1.7 % of patients in the edoxaban and enoxaparin groups, respectively (*P* = 0.769). There was no significant difference in the incidence of major or CRNM bleeding during the treatment period in the edoxaban group (2.6 %) compared with the enoxaparin group (3.7 %; *P* = 0.475). Breakdowns of major and CRNM bleeding events are provided in Table 3. The incidence of any bleeding events (major, CRNM, and minor bleeding) was 20.5 % vs 15.9 % in the edoxaban and enoxaparin groups, respectively (*P* = 0.151). The incidence of minor bleeding events was significantly greater in the edoxaban group compared with the enoxaparin group (*P* = 0.049). The presence of blood in the urine was characterized as minor bleeding, and occurred in 38 patients in the edoxaban group (12.5 %) and 34 patients in the enoxaparin group (11.3 %). No cases of fatal bleeding occurred in either group.

**Table 3:** Incidence of bleeding events during the treatment period

Event	Edoxaban 30 mg QD (*n* = 303)		Enoxaparin 2000 IU BID (*n* = 301)		Absolute difference % (95 % CI)	*P*
	n (%)	95 % CI	n (%)	95 % CI		
Major bleeding	2 (0.7)	0.2, 2.4	6 (2.0)	0.9, 4.3	−1.3 (−3.7, 0.7)	0.176
Subcutaneous hemorrhage	1 (0.3)	NC	2 (0.7)	NC	NC	NC
Wound hemorrhage	1 (0.3)	-	1 (0.3)	-	-	-
Duodenal ulcer hemorrhage	0 (0.0)	-	2 (0.7)	-	-	-
Hemarthrosis	0 (0.0)	-	1 (0.3)	-	-	-
CRNM bleeding	6 (2.0)	0.9, 4.3	5 (1.7)	0.7, 3.8	0.3 (−2.1, 2.8)	0.769
Subcutaneous hemorrhage	1 (0.3)	NC	0 (0.0)	NC	NC	NC
Wound hemorrhage	1 (0.3)	-	0 (0.0)	-	-	-
Conjunctival hemorrhage	1 (0.3)	-	0 (0.0)	-	-	-
Epistaxis	1 (0.3)	-	0 (0.0)	-	-	-
Lower gastrointestinal hemorrhage	1 (0.3)	-	0 (0.0)	-	-	-
Hematuria	1 (0.3)	-	1 (0.3)	-	-	-
Postprocedural hematoma	0 (0.0)	-	3 (1.0)	-	-	-
Subcutaneous hematoma	0 (0.0)	-	1 (0.3)	-	-	-
Major or CRNM bleeding	8 (2.6)	1.3, 5.1	11 (3.7)	2.1, 6.4	−1.0 (−4.1, 1.9)	0.475
Minor bleeding	57 (18.8)	14.8, 23.6	39 (13.0)	9.6, 17.2	5.9 (0.0, 11.7)	0.049
Any bleeding	62 (20.5)	16.3, 25.4	48 (15.9)	12.2, 20.5	4.5 (−1.7, 10.7)	0.151

BID, twice daily; CI, confidence interval; CRNM, clinically relevant nonmajor bleeding; IU, international unit; NC, not calculated; QD, once daily

Patients could have multiple bleeds, which were counted individually

The overall incidence of AEs from the start of treatment to the day of the follow-up examination was 65.0 % (197/303) in the edoxaban group and 77.1 % (232/301) in the enoxaparin group (Table 4). The most common AEs that occurred in ≥5 % of either treatment groups were increased γ-glutamyltransferase, blood present in the urine, increased ALT, increased AST, increased blood alkaline phosphatase, and subcutaneous hemorrhage. Elevated ALT or AST levels ≥3 times the ULN was higher in the enoxaparin group (10.0 %) than in the edoxaban group (2.6 %; Table 4). The pattern of the incidence of ADRs was similar to that of AEs. All AEs and ADRs were mild or moderate in intensity. The incidences of AEs and ADRs excluding bleeding events and hepatic dysfunction were 41.6 % and 11.2 %, respectively, in the edoxaban group, and 44.9 % and 14.0 %, respectively, in the enoxaparin group.

**Table 4:** Adverse events, adverse drug reactions, and abnormal changes in hepatic function

	Edoxaban 30 mg QD (*n* = 303)	Enoxaparin 2000 IU BID (*n* = 301)
**Adverse events**		
Patients with events	197 (65.0)	232 (77.1)
95 % CI	59.5, 70.2	72.0, 81.5
Number of events	448	676
**Adverse events reported by ≥5 % of patients in either treatment group** ^**a**^		
ALT increased	36 (11.9)	126 (41.9)
AST increased	17 (5.6)	97 (32.2)
γ-glutamyltransferase increased	44 (14.5)	79 (26.2)
Blood urine present	38 (12.5)	34 (11.3)
Blood alkaline phosphatase increased	14 (4.6)	40 (13.3)
Hemorrhage subcutaneous	12 (4.0)	21 (7.0)
**Adverse drug reactions**		
Patients with events	121 (39.9)	177 (58.8)
95 % CI	34.6, 45.5	53.2, 64.2
Number of events	198	439
**Hepatic function test parameter**		
ALT or AST		
≥1.5 x ULN	43 (14.2)	135 (44.9)
≥3 x ULN	8 (2.6)	30 (10.0)
≥5 x ULN	2 (0.7)	11 (3.7)

Data are presented as n (%) unless otherwise indicated. ALT, alanine aminotransferase; AST, aspartate aminotransferase; BID, twice daily; CI, confidence interval; IU, international unit; QD, once daily; ULN, upper limit of normal

^a^Classified by MedDRA/J V.12.0 Preferred Term

There was no difference in the incidence of serious AEs (SAEs) between the edoxaban (3.0 %) and enoxaparin (3.0 %) treatment groups. Nine SAEs occurred in the edoxaban group; for all of these, a causal relationship was ruled out by the investigator. In the enoxaparin group, 10 SAEs occurred; for nine of them, a causal relationship was ruled out, excluding one case of postoperative wound infection.

### Plasma concentrations of edoxaban

The PK analysis set included a cohort of 250 patients. However, laboratory anomalies detected after remediation of the bioanalytic results identified 36 samples as inevaluable, leaving 214 patients in the updated PK analysis set. The mean (standard deviation) edoxaban plasma concentration in these 214 patients at predose on day seven of treatment was 17.8 (12.1) ng/mL. The plasma concentration was similar to the results obtained in the original PK analysis set of 250 patients before remediation.

## Discussion

The STARS J-V trial demonstrated the noninferiority and superiority of once-daily oral edoxaban 30 mg to twice-daily subcutaneous enoxaparin 2000 IU for the prevention of VTE in Japanese patients undergoing elective unilateral THA based on venography. Although no symptomatic PE occurred, this was not unexpected, as the rate of symptomatic PE following THA or TKA has a very low event rate of 0.55 % in Japan [16]. The incidence of VTE for patients receiving edoxaban 30 mg has been previously explored in phase 2b studies of patients undergoing TKA or THA [9, 11]. In a study evaluating edoxaban vs enoxaparin in patients undergoing THA in Japan and Taiwan [11], the incidence of thromboembolic events in the edoxaban 30-mg group (2.8 %) was consistent with the current study (2.4 %). In a placebo-controlled study in Japanese patients undergoing TKA [9], VTE occurred in 12.5 % of patients in the edoxaban 30-mg once-daily treatment group, which is higher than the incidence of VTE reported in the edoxaban group in this study. The higher rate of VTE may be explained by an older population in the phase 2b TKA study compared with STARS J-V (mean age 71.4 vs 62.8 years). A recent analysis by Rohatagi, *et al.* [17] explored the exposure vs response relationship between edoxaban and VTE rates using pharmacometric analyses. In addition to a direct relationship between edoxaban exposure and the incidence of VTE, they showed that increasing age is a risk factor for VTE [17].

In this study, the incidences of any bleeding and major or CRNM bleeding events were similar for edoxaban and enoxaparin. In addition, the rates of major or CRNM bleeding events in patients receiving edoxaban 30 mg were low in our study (2.6 %) and similar to the phase 2b studies in TKA and THA (3.9 % and 1.2 %, respectively) [9, 11]. The incidence of any bleeding was higher in our study (20.5 %) compared with that observed in the edoxaban 30-mg treatment group in the phase 2 TKA trial (10.7 %) [9]. Although the rates of major bleeding and CRNM bleeding were low and similar between these studies, the incidence of minor bleeding was high for edoxaban in our study (18.8 %). Reasons for the higher rate of minor bleeding in the edoxaban group are not clear. Higher rates of bleeding may be attributed, at least in part, to the very high rate of NSAID use—98 % of patients in the edoxaban group concomitantly used NSAIDs compared to 81 % in the enoxaparin group. In a retrospective analysis of pooled data from four phase 3 studies evaluating the safety and efficacy of rivaroxaban compared with enoxaparin for the prevention of VTE after THA or TKA, the risk of bleeding increased with concomitant use of NSAIDs in either treatment group [18]. Higher rates of major bleeding following administration of low molecular weight heparin have been reported at 2–4 h postoperatively compared with 12–48 h postoperatively, suggesting that higher levels of anticoagulation earlier may be associated with more bleeding events [19]. As edoxaban was initiated with an earlier start than enoxaparin, this may contribute to the higher observed rates of minor bleeding. Lastly, no fatal bleeding or bleeding of critical sites (eg, intracranial bleeding) occurred in this study.

The incidence of all AEs in this study was lower in the edoxaban group than in the enoxaparin group. In addition, elevations in ALT or AST were seen in fewer patients in the edoxaban group vs the enoxaparin group. However, it should be noted that elevated serum transaminase levels are recognized as a class effect of heparins. These elevations are transient and reversible and considered benign [20]. Overall bleeding events were more common with edoxaban. The AEs other than bleeding events and AEs related to abnormal hepatic function test values were similar between both treatment groups. In addition, the incidence of SAEs was similar in both the edoxaban and enoxaparin groups.

Other oral direct factor inhibitors have been compared with subcutaneous enoxaparin 40 mg for thromboprophylaxis following elective hip replacement and have also been shown to effectively reduce the risk for VTE postsurgery without increasing the risk for clinically relevant bleeding [21–23]. However, in these studies, only 0 %–6.8 % of randomized patients were Asian. It should be noted that in the current study, the Japanese patient population was smaller than the population in the other THA studies, which had enrolled patients from Western countries. Additionally, the dose of enoxaparin used (2000 IU, twice daily) is a recommendation specific to Japan for the prevention of VTE [4]. Japanese patients typically have a lower body weight than patients from the United States or the European Union; notably the mean body weight in this study was 57 kg compared with a mean body weight of 78 kg for patients in the phase 3 trial for rivaroxaban in THA, which included primarily white patients (92 %) [21]. A body weight for males >57 kg is associated with increased enoxaparin exposure and increased bleeding risk for prophylactic, subcutaneous enoxaparin doses of 40 mg once daily or 30 mg twice daily [24]. Thus, these results are primarily relevant to Japanese patients.

In conclusion, the STARS J-V study demonstrated the superiority of oral once-daily edoxaban 30 mg compared with twice-daily subcutaneous injection of enoxaparin 2000 IU for the prevention of VTE in Japanese patients after THA, without an increased risk of bleeding or AEs. The favorable efficacy-to-safety balance of edoxaban suggests an attractive option for thromboprophylaxis in patients following THA.

## Appendix

Sponsor’s Medical Experts − S. Tachibana, M. Nakamura, Y. Koretsune, T. Yamashita. The event adjudication committee and investigators are as follows: Data Monitoring Committee − H. Origasa, M. Murata, S. Yanagimoto; Thromboembolic Event Assessment Committee − H. Nakamura, K. Nakamura, S. Fujita, S. Nakata; Bleeding Event Assessment Committee − S. Fujita, Y. Kawai; Study Investigators − M. Tsuji, K. Kitamura, T. Masuda, J. Ito, T. Sano, K. Sato, K. Kawamae, T. Kikuchi, M. Saito, T. Kodama, T. Matsuda, T. Sasaki, S. Hasegawa, K. Maeda, N. Mitsugi, T. Mori, K. Uchiyama, A. Hyodo, S. Kondo, T. Matsumoto, K. Suzuki, T. Tani, S. Iwasada, O. Obayashi, S. Kitamura, T. Sato, T. Yamakawa, K. Ogawa, H. Iwaki, J. Tamura, S. Osawa, H. Ohashi, K. Fujiwara, M. Kakiuchi, K. Nakata, H. Kagiyama, S. Kushitani, S. Saito, H. Tokunaga, H. Iida, N. Shibanuma, T. Nakai, T. Matsubara, N. Nakagawa, H. Kishimoto, N. Ikeda, Y. Nanba, T. Sato, M. Tsubouchi, J. Nakashiro, T. Tani, Y. Ito, M. Naito, Y. Esaki, F. Higuchi, Y. Doiguchi, K. Yonemori, M. Nagayama; Sponsor Representatives − T. Kimura (Project Leader), M. Fukuzawa (Study Leader), K. Abe (Statistician).

## Abbreviations

ADR: Adverse drug reaction
AE: Adverse event
ALT: Alanine aminotransferase
aPTT: Activated partial thromboplastin time
AST: Aspartate aminotransferase
CI: Confidence interval
CRNM: Clinically relevant nonmajor
DVT: Deep vein thrombosis
FAS: Full analysis set
INR: International normalized ratio
LMWH: Low-molecular weight heparin
PD: Pharmacodynamics
PE: Pulmonary embolism
PK: Pharmacokinetics
PPS: Per-protocol set
PT: Prothrombin time
SAE: Serious adverse event
SAS: Safety analysis set
STARS J-V: Studying Thrombosis After Replacement Surgery J-V
THA: Total hip arthroplasty
TKA: Total knee arthroplasty
VTE: Venous thromboembolism
